# First report on HLA*-DPA1* gene allelic distribution in the general Lebanese population

**DOI:** 10.1016/j.mgene.2016.01.004

**Published:** 2016-01-21

**Authors:** Joseph Haddad, Dina Shammaa, Fatmeh Abbas, Rami A.R. Mahfouz

**Affiliations:** Department of Pathology & Laboratory Medicine, AUBMC, Lebanon

**Keywords:** DPA1, Locus, Allele, Frequency, Lebanon

## Abstract

**Aims:**

HLA-*DPA1* is an important marker in bone marrow and organ transplantation and a highly emerging screening parameter in histocompatibility laboratories. Being highly polymorphic, it has another significant value in detecting population origins and migrations. This is the first study to assess *DPA1* allele frequencies in an Arab population.

**Methods:**

The HLA *DPA1* alleles were identified using the One-Lambda assays on a Luminex reverse SSO DNA typing system. Our study included 101 individuals coming from different Lebanese geographical areas representing the different communities and religious sects of the country.

**Results:**

We compared the results of this study to 16 different populations and found very interesting similarities and differences between Lebanese people and individuals of European ancestry.

**Conclusion:**

This study is the first to describe the different allelic frequencies of HLA-*DPA1* in the Lebanese population and will serve as a template that can be later used for disease association studies both at the level of the country and internationally.

## 1 Introduction

Human leukocyte antigen (HLA) compatibility is necessary for a desirable organ/stem cell transplantation and tests performed on donors and recipients determine the extent of this compatibility. A perfect or near-perfect HLA match is necessary to classify a transplant as successful or not. Identifying the desired HLA locus responsible for an engraftment is essential for optimal HLA matching to minimize graft-versus-host-disease and engraftment failure (Morishima et al., 2015). HLA-*DP* represents one of the three major MHC class II proteins encoded (HLA-*DQ* and HLA-*DR*). These HLA groups of genes are found on the short arm of human chromosome 6.

HLA*-DP* encodes for the *DP* antigen on the surface of cells and has been associated with a lower immunostimulatory effect than HLA*-DQ* and HLA*-DR* (Varney et al., 2010). The other HLA groups also encode different proteins that are present on the surface of cells. Hence, the whole three major classes determine the ability of a donor's tissue or organ to be accepted by a recipient. HLA-*DQ* and HLA-*DR* have shown to be the main players in controlling graft rejection or acceptance, yet the *DP* locus has its share in deciding the final outcome.

HLA-*DP* is made of two subunits, alpha (*DPA*) and beta (*DPB*). Each subunit is encoded by its corresponding locus; DPα by *DPA1* and DPβ by *DPB1*. The *DPB1* locus is more variable and polymorphic than the *DPA1* locus (Petersdorf et al., 2001). However, accounting for this limited polymorphism (compared to *DPB1*) is essential for survival rates and graft acceptance. HLA-*DPA1* mismatched stem cell graft patients showed reduced survival rates and shorter recurrence-free survival rates (Schaffer et al., 2003). Moreover, in accordance with its importance, the effect of HLA-*DPA1* was investigated in various clinical diseases such as inflammatory bowel disease (Lantermann et al., 2002), post-streptococcal acute glomerulonephritis (Mori et al., 1996), ankylosing spondylitis (Díaz-Peña et al., 2013), systemic lupus erythematosus and systemic vasculitis (Gan et al., 2015). It was also studied in seropositive chronic hepatitis B patients on IFN treatment (Cheng et al., 2014).

Due to HLA-*DPA1* and HLA-*DPB1* variability and polymorphism, these genes can vary greatly among different ethnic groups. Hence, they are of great importance in human immunogenetics and in providing information about human population diversity. These serve as important markers in detecting population origins and migrations. Since Lebanon is a country that falls between Europe and Asia, its soil was stepped upon by many populations. Some left, some stayed. Hence Lebanon is looked at as a mixture of different populations, which confers the existence of different *DPA1* alleles.

The aims of this study are to detect the different HLA-*DPA1* alleles in the Lebanese population (Aldener-Cannavá and Olerup, 1996) and to compare them to the published literature in different countries such as the Basque population in Spain (Begovich et al., 2001, Pérez-Miranda et al., 2004), Caucasoid (Swedish) (Cheng et al., 2014) and West African (Gambia) (Díaz-Peña et al., 2013) populations (Aldener-Cannavá and Olerup, 1996), Caucasoid (British) (Gan et al., 2015) population (Sage et al., 1994), Japanese (Harada et al., 1992), Papua New Guinean (Hollenbach et al., 2012), and Buyi-Chinese (Khansa et al., 2012) populations (Harada et al., 1992), four Pacific islands (Khansa et al., 2013, Lantermann et al., 2002, Mori et al., 1996, Morishima et al., 2015) populations (Cook Islands (Khansa et al., 2013), Samoa (Lantermann et al., 2002), Tokelau (Mori et al., 1996), Tonga (Morishima et al., 2015); (Velickovic and Carter, 2001), Guadeloupe (Pérez-Miranda et al., 2004) population (Voorter et al., 2014), Cameroon (Petersdorf et al., 2001), Ecuadorian African (Sage et al., 1994), and Indonesian (Schaffer et al., 2003) populations (Begovich et al., 2001), and individuals of European ancestry (Shammaa et al., 2010, Hollenbach et al., 2012). This study is the first to describe the different allele frequency in the Lebanese population and can be later used for disease association studies.

## 2 Materials and methods

### 2.1 Samples

We analyzed the reported HLA results of samples from 101 individuals referred for HLA *DPA1* typing at the Histocompatibility and Molecular Diagnostics Laboratory of the American University of Beirut Medical Center. These individuals are patients and potential bone marrow donors (screened by their referring attending physicians and transplant specialists), who come from different Lebanese geographical areas and communities, and are part of a National HLA Registry at our institution. However, for this study, only donors were included and they are all unrelated.

### 2.2 DNA extraction

DNA was extracted by the automated DNA extraction technique using the QiaCube kit (Qiagen, California, USA). The extracted products were properly labeled and stored at − 80 °C.

### 2.3 HLA-*DPA1* genotyping

The HLA-*DPA1* alleles were identified using the Luminex reverse SSO DNA typing system (LABScan 100™, xPONENT, Powered by Luminex xMAP Technology, USA) which consists of sequence specific oligonucleotide probes bound to color-coded microspheres. The extracted DNA is first amplified with biotinylated primers using the polymerase chain reaction (PCR). The amplification is done using the following program: initial denaturation at 96 °C for 3 min followed by 5 cycles of denaturation at 96 °C for 20 s, annealing of probes at 60 °C for 20 s, and extension of DNA at 72 °C for 20 s. This step is followed by 30 cycles of denaturation at 96 °C for 10 s, annealing of probes at 60 °C for 15 s, and extension of DNA at 72 °C for 20 s. The final step includes the temperature being set at 7 °C for 10 min. Finally, temperature is set at 4 °C until product is removed. The amplification is verified by electrophoresis on a 2% agarose gel stained with ethidium bromide. The PCR product is biotinylated, which allows it to be detected using R-Phycoerythrin-conjugated Streptavidin (SAPE). Reading on the Luminex analyzer was then performed following the manufacturer's instructions.

## 3 Results

The frequencies of the various HLA-*DPA1* alleles reported in this study are listed in Table 1 where results are compared with the frequencies of several alleles reported in different populations labeled from 1 to 17. The allelic frequencies in the chosen Lebanese sample were calculated by direct counting method and reported as percentages. The detected alleles in the Lebanese population are DPA1*0103, DPA1*0201, DPA1*0202, and DPA1*0301 with allelic frequencies of 86.14%, 9.9%, 2.97%, and 0.99% respectively.

## 4 Discussion

HLA-*DP* represents one of the three major MHC class II loci (including also HLA-*DQ* and HLA-*DR*) that are found on the short arm of chromosome 6. The HLA-*DP* locus has a lower immunostimulatory effect than the other two HLA groups of genes (Varney et al., 2010). However, it is still an important player in bone marrow and organ transplantation and can affect graft survival as well as outcome of the transplant. HLA-*DP* has two subunits of alpha and beta encoded by HLA-*DPA1* and HLA-*DPB1* respectively. Both are highly polymorphic, with HLA-*DPB1* being slightly more variable (Petersdorf et al., 2001). Being so, *DPA1* and *DPB1* can be used to study origins and migrations of different populations from all over the world. This study is the first of its kind that has assessed HLA-*DPA1* typing in the Lebanese community with the frequencies of 9 different HLA-*DPA1* investigated and compared with those available from 16 healthy ethnic groups.

The most predominant allele in our study population was DPA1*0103 with a prevalence rate of 86.14% which is very similar in being the highly prevalent population-specific allele for all of the European countries and populations included in this study like the Basque population in Spain (Begovich et al., 2001), Caucasoid (Swedish) (Cheng et al., 2014), and individuals of European ancestry (Shammaa et al., 2010), in addition to Tokelau of the Pacific Islands (Mori et al., 1996). This information is pertinent if we mention here the historical fact about Lebanon being previously under the influence of the Crusaders invasion of the region with all inter-marriages that took place among different populations and communities then.

Asian countries like the Buyi-Chinese (Khansa et al., 2012), Japanese (Harada et al., 1992), and Indonesians (Schaffer et al., 2003) have a relatively low prevalence of DPA1*01 when compared to our Lebanese population (Aldener-Cannavá and Olerup, 1996), but with DPA1*0202 as the predominant allele. Our study showed that the Lebanese population has a DPA1*0202 prevalence of 2.97%, which is very low when compared to the latter populations with more than 30% prevalence rate.

The African countries studied were the West Africans (Gambia) (Díaz-Peña et al., 2013), Cameroon (Petersdorf et al., 2001), and Ecuadorian Africans (Sage et al., 1994) which showed a relatively high prevalence of the DPA1*0201 allele, and is thus considered their predominant allele (with a prevalence of at least 22%) when compared to the Lebanese population (9.9%).

The four Pacific islands (Khansa et al., 2013, Lantermann et al., 2002, Mori et al., 1996, Morishima et al., 2015) listed almost show an equal prevalence between the DPA1*0103 and DPA1*0202 alleles (with the Tokelau (Mori et al., 1996) population being an exception and showing much higher DPA1*0103 prevalence). The Guadeloupe (Pérez-Miranda et al., 2004) study population shows DPA1*0103 to be its predominant allele (38%) with a close prevalence rate of 30% for DPA1*0201. These populations' prevalence rates are very divergent from our reported Lebanese population data.

In light of these results, the similarity between the studied Lebanese population and individuals of European ancestry (Shammaa et al., 2010) was most appealing. The descending order of percentages in terms of most to least frequent allelic variations is remarkably similar. Respectively, the Lebanese population showed prevalence rates of 86.14%, 9.9%, 2.97%, and 0.99% and descendants of European ancestry showed 81.86%, 14.03%, 3.43%, and 0.11% for DPA1*0103, DPA1*0201, DPA1*0202, and DPA1*0301 alleles.

Previous studies conducted on different HLA classes, loci, and alleles revealed significant relationships to Europeans as well. The study conducted on HLA class II allele frequencies in the Lebanese population (Khansa et al., 2012), HLA-*DQA1* gene allelic distribution (Shammaa et al., 2011), in addition to HLA-*DPB1* gene allelic distribution (Shammaa et al., 2010), and HLA class I allelic frequencies (Khansa et al., 2013) in the Lebanese population show significant genetic relations to Europeans; specifically, Bulgarian, Polish, Spanish, Greek, Irish, and Swiss populations.

Finally, Lebanon's geographical location in the Middle East opened many European rulers' eyes to take over Lebanon in order to gain control of its strategic standpoint into Asia. From Alexander the Great, to the French colonization in the 1930s, Europeans played a major role in this part of the world. Many stayed and blended with the Lebanese population, leading to the close genetic similarity in *DPA1* alleles between the Lebanese and the European populations. This first report in the Lebanese population will be an important source of information for other studies in the region as well as an added asset to national and international bone marrow stem cell transplantation programs seeking the best matching donors for the corresponding patient.

## Figures and Tables

**Table 1:** Allelic frequency of the DPA1 alleles in different populations including the Lebanese community (current study).

	N (sample size)	DPA1*01:03	DPA1*01:04	DPA1*01:05	DPA1*02:01	DPA1*02:02	DPA1*02:03	DPA1*03:01	DPA1*03:02	DPA1*04:01
Lebanon (Aldener-Cannavá and Olerup, 1996) (current study)	101	86.14	0	0	9.9	2.97	0	0.99	0	0
Basque population (Spain) (Begovich et al., 2001)	116	76.72	NR	1.72	18.53	2.16	0.43	0.43	NR	NR
Caucasoid (Swedish) (Cheng et al., 2014)	100	86.5	0	NR	7	6	NR	0.5	NR	0
West Africans (Gambia) (Díaz-Peña et al., 2013)	100	32.5	0	NR	50.5	8.5	NR	8.5	NR	0
Caucasoid (British) (Gan et al., 2015)	187		78.1		17.4	4.3	NR	0.3	NR	0
Japanese (Harada et al., 1992)	227		38.5		18.1	43.4	NR	0	NR	0
Papua New Guinea (Hollenbach et al., 2012)	88		86.9		3.4	9.1	NR	0	NR	0.6
Buyi- Chinese (Khansa et al., 2012)	41		19.5		3.7	70.7	NR	0	NR	6.1
Cook Islands (Khansa et al., 2013)	50	54	NR	NR	0	44	NR	NR	NR	2
Samoa (Lantermann et al., 2002)	50	42	NR	NR	1	57	NR	NR	NR	0
Tokelau (Mori et al., 1996)	50	73	NR	NR	1	26	NR	NR	NR	0
Tonga (Morishima et al., 2015)	50	46	NR	NR	0	53	NR	NR	NR	1
Guadeloupe (Pérez-Miranda et al., 2004)	154	37.98	0.32	0.32	30.19	17.53	NR	12.33	0.97	0.32
Cameroon (Petersdorf et al., 2001)	172	37.2	0	NR	22.7	9.6	NR	26.2	1.7	3.2
Ecuadorian Africans (Sage et al., 1994)	58	20.7	0.9	NR	43.1	21.6	NR	13.8	0	0
Indonesians (Schaffer et al., 2003)	132	23.1	0	NR	15.9	56.8	NR	0	0	4.2
European ancestry (Shammaa et al., 2010)	5944	81.86	0.53	0.01	14.03	3.43	0.01	0.11	0.01	0.01

NR = Not Reported.
